# A crystal-clear path to promoting crystallography to undergraduate students

**DOI:** 10.1063/4.0000974

**Published:** 2025-10-27

**Authors:** Carlos A Marrufo, Susanna Huang Undergraduate

**Affiliations:** Georgia Institute of Technology, Atlanta, GA, USA

## Abstract

The Structural Nucleic Acid Anticancer Research Society, otherwise known as STARS, is a nonprofit organization that is founded on showcasing crystallography to K-12th and undergraduate students in an effort to grow student interest in the subject. With passionate officers and great leadership, the organization has expanded greatly over the years through outreach programs and club branch activities. The STARS mission is to share with students the importance of structural biology for therapeutic drug discovery and to provide students opportunities to explore what crystallography has to offer in research.

The STARS Atlanta Branch at GT specifically provides crystallography workshops, skills-building events, and research lectures to provide students with the opportunity to learn valuable scientific skills and obtain research career insight. In 2024 alone, STARS organized a total of five crystallography workshops for undergraduate and high school students. At our workshops, we taught students how to crystallize lysozyme proteins and gave them insightful lectures on crystallography research, providing exciting opportunities for students to not only learn valuable scientific skills, such as micro pipetting and setting up their own experiments, but also be introduced to crystallography as it relates to therapeutic drug design. We utilize these outreach programs as outstanding opportunities to introduce new students to both the research field and our STARS nonprofit branch.

Besides our outreach events, we organize weekly branch meetings to engage routine STARS student members in crystallography. In these STARS meetings we provide students the chance to crystallize proteins to give students wet-lab and dry-lab research adjacent experiences, such as learning how to read literature, how to set up experiments, and how to analyze their results. By exposing the students to these opportunities, they can strengthen their scientific skills and intuition for their future research endeavors. Specifically for computational research experience, our organization has helped develop students' skills in model building and refinement by teaching them how to begin using PHENIX and COOT. Students were shown and taught the protein model building and refinement process to obtain more accurate protein structures that matched the experimental data. PHENIX and COOT helped our students visualize the protein and protein complex structures that could be studied and analyzed through crystallography. To further expand the knowledge of our STARS GT branch’s members, we even had the opportunity to visit the Oak Ridge National Laboratory (ORNL) in October of 2024. We aimed to use this trip to show our students the different types of crystallography research done and their importance in a government laboratory. In a more local tour, our STARS GT branch had the chance to participate in a tour of the La Pierre lab at Georgia tech, which helped show students what crystallography research can be like in a research lab setting. In the near future STARS aims to provide more therapeutically relevant crystallography projects to its students, such as crystallizing DNA with their protein complexes to understand their intermolecular interactions. These macromolecular crystallography experiments can be outstanding opportunities for students to experience and learn in extracurricular settings how research can be proposed, executed, and analyzed for important research questions, providing them important and lasting scientific skills which they can use for their own research endeavors.

